# Supplementary feeding affects the breeding behaviour of male European treefrogs (*Hyla arborea*)

**DOI:** 10.1186/1472-6785-9-1

**Published:** 2009-01-07

**Authors:** Ivonne Meuche, T Ulmar Grafe

**Affiliations:** 1 Department of Animal Ecology and Tropical Biology, Biozentrum, University of Würzburg, 97074 Würzburg, Germany; 2 Department of Biology, Universiti Brunei Darussalam, Tungku Link, Gadong BE 1410, Brunei Darussalam

## Abstract

**Background:**

We investigated the effects of energetic constraints on the breeding behaviour of male European treefrogs *Hyla arborea *and how calling males allocated additional energy supplied by feeding experiments.

**Results:**

Presence in the chorus was energetically costly indicated by both fed and unfed males losing weight. Males that were supplied with additional energy did not show longer chorus tenure. Instead, fed males returned sooner to the chorus. Additionally, fed males called more often than control males, a novel response for anurans. A significantly higher calling rate was noted from males even 31 nights after supplementary feeding.

**Conclusion:**

This strategy of allocating additional energy reserves to increasing calling rate is beneficial given the preference of female hylids for males calling at high rates and a female's ability to detect small incremental increases in calling rate.

## Background

How organisms acquire resources and allocate them to the demands of maintenance, defence, repair, storage, growth and reproduction are central questions in physiological ecology [1]. After energy and nutrients have been partitioned between these major demands, further allocations can be made. For example, investing in reproduction will vary according to age and energetic status with important fitness consequences. Numerous studies have reported morphological and behavioural attributes influencing male mating success that include display rates [2], body size [3-6], body condition [7,8], lek centrality [5,9] and lek attendance [10-13]. Many of these attributes reflect how males can acquire energy and how they allocate it during the reproductive season.

Male anurans are especially interesting subjects to study how energy is partitioned for reproduction, because calling is energetically very expensive [14] and acoustic energy can easily be partitioned between call duration, calling rate, call amplitude, number of hours calling within a night, number of nights calling within a breeding season, and the number of breeding seasons in attendance.

In anurans, inter- and intrasexual selection are important determinants of mating success [15,16]. In most species of anurans with a lek mating system studied to date, male mating success is determined by the number of nights that a male is present in a breeding aggregation, i.e. chorus tenure [17]. Thus, there should be strong selection on males to increase their chorus tenure. Paradoxically, males of most anuran species spend less than 20% of the breeding season in the chorus [17,18].

A variety of hypotheses have been suggested that might explain short chorus tenure in anurans [13,19]. First, high rates of mortality within and away from the chorus are one hypothesis. Second, chorus tenure could be underestimated if males move between ponds and monitoring is restricted to a single chorus. The energetic limitation hypothesis offers a third explanation for short chorus tenure in anurans. Males may need to allocate the available energy into the maintenance of vital processes and growth with limited energy reserves left for reproduction and attracting females. Since calling is energetically very costly [14], males will not be able to maintain high rates of calling, or be present in the chorus on many nights, conditions that are necessary to attract females.

So far, studies of energy allocation in anurans have concentrated on the effects of energetic constraints on body condition and chorus tenure. Short term allocation strategies such as modulating the number of calls produced during nightly chorus attendance or varying calling rates have received less attention [20-22]. If calling rate and chorus tenure of a male are positively correlated with his reproductive success, there should be a trade-off between both parameters since males will be unlikely to be able to allocate unlimited energy to both behaviours. Males should allocate energy preferentially to the strategy that most strongly affects male mating success.

In this paper we investigate the effects of energetic constraints on breeding behaviour of male European treefrogs, *Hyla arborea *(Linnaeus, Anura: Hylidae), a species of considerable conservation interest [23,24]. We hypothesized that if breeding behaviour is energetically limited, then males should lose body condition between their first and last night in the breeding chorus. If additional energy is provided experimentally, then fed and unfed males should have different final conditions and different rates of change in condition. If the prime target of selection is to increase chorus tenure, then males provided with food should be in the chorus more nights than unfed males. In addition, fed males should return to the chorus sooner than unfed males. If the prime target of selection is to call at high rates and thus out-compete other males, then fed males should allocate the available energy to calling on few nights and should have a higher rate of calling than unfed males.

## Results

### Body condition

The mean snout-vent length and tibiafibula length of captured males was 41 ± 2 mm (N = 45) and 21 ± 1 mm (N = 45), respectively. The average initial weight was 5.65 ± 0.78 g. The median initial body condition was 0.09 g for Fed1, 0.05 g for Fed2 and -0.04 g for the control males. Males in the three groups did not differ in their initial body condition (Kruskal-Wallis: H = 0.8, N = 43, p > 0.05).

No significant relationship was detectable between the first noted presence at the pond and the condition that same day (Pearson correlation: r = -0.21, N = 44, p > 0.05). However, the control males (paired t-test: T = 2.47, N = 16, p < 0.05) as well as Fed1 males (paired t-test: T = 2.81, N = 8, p < 0.05) and Fed2 males (paired t-test: T = 3.48, N = 10, p < 0.01) lost weight significantly during the season (Table 1). The average final condition was not influenced by feeding (Table 1). Fed as well as unfed males were in the same condition at the end of the season (ANOVA: F = 0.28, N = 34, p > 0.05). In addition, the supply of supplementary energy showed no influence on the rate of change in body condition (Kruskal-Wallis: H = 1.29, N = 35, p > 0.05; Table 1).

### Chorus tenure

**Figure 1:**
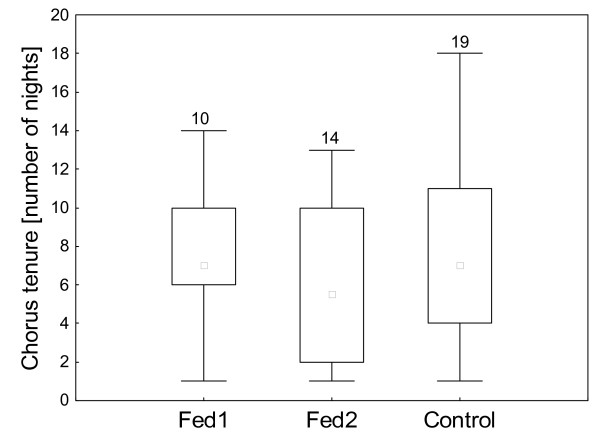
**Male chorus tenure**. Chorus tenure of males according to their treatment group (Fed1; Fed2; Control) in the year 2002 (medians, 1. and 3. quartile); the values insight the figure indicates the sample size.

In 2002, chorus tenure varied between the individual males. The median chorus tenure for Fed2 was 5.5 nights; for the control males and Fed1 the median chorus tenure was 7 nights (Fig. 1). Thus, both the control males and Fed1 males were present in the chorus for only 18.4% of the possible time. Fed2 spent only 14.5% of the nights in the chorus. Five males were only present 1 night (one Fed1-, two Fed2- and two control males). On average, control males stayed 19.2 nights in the calling aggregation (first until final night). This time interval amounted to 19.0 nights for Fed1 and to 12.3 nights for Fed2.

There were no significant differences in chorus tenure between the treatment groups (Anova: F = 0.617, N = 42, p > 0.05). Males, which were fed, did not return to the chorus for more nights compared to males that were not provided with extra food.

Among the fed males (Fed1 and Fed2 pooled) the mass of consumed crickets (and thus the quantity of energy taken in) did not explain chorus tenure (linear regression: r^2 ^= 0.054; T = -1.1, N = 23, p > 0.05). Males that had consumed more energy did not stay in the chorus longer than males that had taken up less energy.

**Figure 2:**
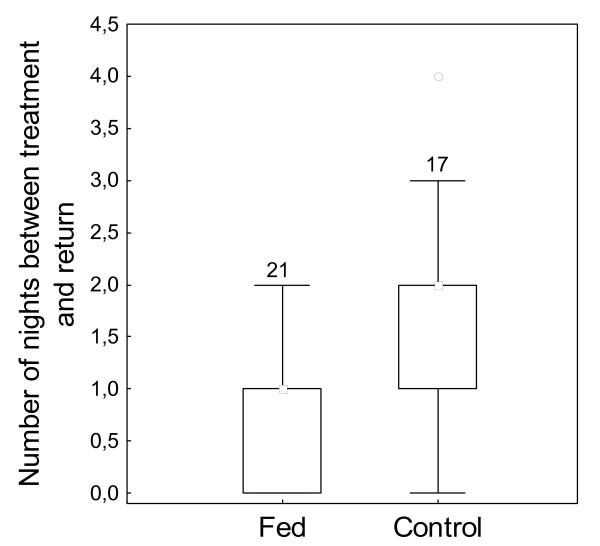
**Number of nights between treatment and return**. Number of nights between the treatment night of the males and the first night, they returned to the chorus in 2002 according to their treatment group (Fed1 + Fed2 (Fed); control); median, 1. and 3. quartile and also outlier are shown; the values insight the figure indicates the sample size.

For fed males, the median number of nights between the treatment night and the first night that they returned was 1. Control males were absent longer (median = 2). Fed males returned back into the chorus after a significantly shorter time (Mann-Whitney U-test: N_1 _= 21, N_2 _= 17, U = 109, p < 0.05; Fig. 2).

### Calling rate

**Figure 3:**
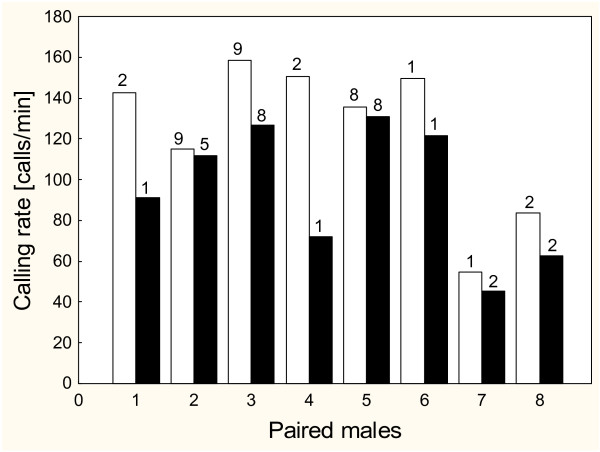
**Calling rate**. Calling rates of paired males according to their treatment group (white bars: Fed1 and Fed2 (Fed); black bars: Control); the numbers indicates the chorus tenure of the males until recordings were taken.

Additional available energy was invested into the rate of calling. Control males had a significantly lower calling rate than fed males (Fed1 and Fed2 pooled; paired t-test: N = 8, T = -3.1, p < 0.05; Fig. 3). This difference in calling rate was shown despite the often long delay (1 to 31 nights) between call recordings and feeding treatment. In 2002, the recordings were taken between a time period of 1 to 31 nights (median = 21 nights) after the treatment; in 2003 they were taken 1 to 2 nights (median = 1.5 nights) after the treatment.

Control males that entered the analysis called at a rate of 95.5 ± 32.3 calls/min, whereas fed males called at 124 ± 36.9 calls/min. Fed1 males received a median of 0.078 g crickets. With an assimilation efficiency of 0.76 [25] this corresponds to a median energy input of 463.62 J. With an average weight of 5.6 g and an average oxygen consumption during calling of 1.076 ml/g·h [20] a call corresponds to 0.0157 J (conversion: 1 litre of used up O2 = 20.08 KJ [26]. With a mean calling rate of 102.2 calls/min males took up enough energy for a further 29,400 calls. Fed2 males consumed a median of five crickets corresponding to 0.25 g, whereby they received sufficient energy for an additional 94,250 calls.

### Mating success

During the reproductive season we observed six pairs in amplexus. Two fed males and two control males paired once and one fed male paired twice. Within this representative sample, the mating success of a male was positively correlated with his chorus tenure (Spearman rank correlation: r_S _= 0.51, N = 44, p < 0.001).

## Discussion

For male tree frogs *Hyla arborea*, the number of nights they stayed within the chorus was a crucial factor for their mating success. However, males showed an short chorus tenure [24,27,28], which is typical of most anurans [13,17,29]. At first the results seem paradoxical. If males can increase their mating success by an increased chorus tenure, why then are they absent during the largest part of the reproductive season? As a possible explanation we tested the hypothesis of energetic limitation. Under this hypothesis males would exhibit reduced chorus tenure, because they are not able to compensate the energetic costs of calling (i.e. by foraging).

As in many other anurans [14] chorus attendance was energetically expensive for male European tree frogs [20]. Males lost significant mass during the investigation period. On average, they lost 0.12 – 0.41 g between their first and last night in the chorus. With a mean weight of 5.65 g males lost between 0.12 – 0.53% of their body mass each night. But fed and unfed males did not differ in final condition or in the rate of change in condition. These results show that males do not invest the energy gained by supplementary feeding into maintaining or establishing energy reserves.

*H. arborea *males can reach momentary rates of oxygen consumption 41-times resting rate during calling [20]. These are the highest aerobic scopes measured so far in any ectothermic vertebrate. The high energetic cost of calling forces males to trade-off call duration, calling rate, call amplitude, number of hours calling within a night, number of nights calling within a breeding season, and the number of breeding seasons in attendance. Our study shows, that male *H. arborea *invest additional energy obtained from food in two ways: they return to the chorus sooner and increase their calling rate.

As in this study, similar feeding experiments of *Rana catesbeiana *[30], *Rana clamitans *[31] and *Physalaemus pustulosus *[32] showed no increase in male chorus tenure. In contrast, Murphy [33] found an increased chorus tenure due to supplementary feeding in *Hyla gratiosa*. In *Hyla arborea*, the energy input seems to have had a short term effect on a male's presence at the pond. Fed males returned back to the chorus after significantly fewer nights than control males suggesting that fed males were able to recover from calling activity sooner.

Calling rate is an important determinant of female choice in most anurans [15,16]. In behavioral tests, females of most anuran species that have been tested prefer males that call at high rates. This preference is generally robust even under acoustically complex field conditions (reviewed by [34]).

If males can increase their reproductive success by calling at higher rates, they should do so if they have sufficient energy reserves. *Hyla arborea *is the first anuran known to allocate supplementary food to calling rate. Males which were supplied with additional energy by supplementary feeding, showed significantly higher calling rates than the control males (Fig. 3). Fed males showed an average calling rate of 124 calls/min, whereas control males produced an average of 95.5 calls/min. It is remarkable, that this difference concerning the calling rate was still detected several days after feeding. In the first year of the study, calling rate was recorded between the 1st and 31st night after males had been fed. In all cases, males seemed to allocate the additional energy in increased calling rate over a period of many nights instead of investing all the energy immediately during the first few nights following feeding.

This strategy of measured energy allocation provides males with the ability to call at higher rates than their competitors and thus remain attractive over many nights. Females of other hylids have been shown to prefer males calling at high rates [15]. A large difference in calling rate in comparison to competitors, however, does not translate proportionally to mating success. In *Pseudacris crucifer *[35] and *Hyperolius marmoratus *[36] females are able to discriminate differences in calling rate of just 12 calls/min (16% difference) and 7 calls/min (15.6% difference), respectively. Additional increases in calling rate did not further increase female preferences. Selection should therefore favour the strategy of a slightly increased calling rate (compared to any competitors), which could be kept up not only for one night but several [37].

In our study, the difference in calling rates of fed males and control males with the same total chorus tenure suggests a trade-off between calling effort and chorus tenure. In this context it would be interesting to determine if and to what degree higher calling rates are preferred by female European tree frogs.

Friedl [38] was not able to show a correlation between the mating success of *Hyla arborea *males and their calling rate. But his method of determining reproductive success was based on the assumption that females can choose between all males present in the chorus. This is highly unlikely and unprecedented. Most likely, females show selective attention for a subset of the males present to minimize the costs of mate sampling thus reducing predation pressure, time, energy, and opportunity costs [39-42]. Such a comparative mate sampling behaviour by females is described by Friedl & Klump [43] in the course of their field observations of *H. arborea*, whereby the females seemed to assess only a few males before they made their mating decision. Additionally, the results of Friedl [38] could be due to male density as well as spatial and temporal pattern in their study population. Above all, if the effect is small, it may be impossible to demonstrate without big sample sizes and multiple year studies. The fact that fed males showed higher calling rates suggest that males would on average benefit from higher mating success at least over evolutionary time.

## Conclusion

Our study suggests that male calling rate is an important criterion of female mate choice. Males invested the additional energy gained by feeding into increasing calling rates. Furthermore, males showed higher calling rates in larger choruses (Meuche & Grafe unpublished) probably due to competition with other males to attract females. In this context, it would be important for future investigations to determine which mate sampling tactic female European treefrogs are using as well as which and to what degree parameters such as calling rate are preferred.

## Methods

### General methods

We studied *Hyla arborea *in southern Germany (Steigerwald) (10.6°E, 49.8°N; 480 m above sea level). Two ponds, formerly but no longer used for raising carp, served as the main study site. Other potential breeding sites were more than 5 km away. There was a distance of 30 m between the two study ponds favoured by the males. They were monitored for 2 years (from 2002 to 2003) whereas only in the year 2002 the presence of males was checked systematically. We checked on arriving and departing treefrogs by installing a drift fence that completely encircling the shore line of the smaller pond with the highest activity. The fence was a slightly modified version of the one used by Murphy [44]. Preliminary tests showed that the fence was an impenetrable barrier for *Hyla arborea *[28]. The fence was patrolled every 5 – 10 minutes. At the same time, the surrounding area of the first pond and especially the second pond (which had no fence) were systematically checked for calling males. With this method, it was possible to identify 71% of calling males attending the second pond each night. Here the checks on calling males started 12 days after the start of the investigation. For most males the calling activity was restricted to just one pond [28]. The breeding period lasted for 54 nights in 2004 (2nd May – 25th June). The main investigation period (14th of May – 25th of June) lasted over 43 nights, excluding five days during which no data was recorded.

A total of 47 different males were caught and marked individually. For marking, both the conventional method of toe clipping and the implantation of the VIAlpha tags (Soft Visible Implant Alphanumeric tags) of Nortwest Marine Technology Inc. (Shaw Island WA, USA). were used. These pliable, fluorescent VIAlpha tags (1.0 × 2.5 mm) were injected laterally under the frog's skin. They have a coding scheme of three alphanumeric characters and a fluorescent orange background. Detection is enhanced with UV-Light. Because the tags are made from a biocompatible medical trade elastomer they do not irritate the tissue at the implant site. VIAlpha tags were successfully tested in a number of frog species [45]. With regard to toe clipping, we only marked two toes or fingers per frog and only one toe or finger per limb. Furthermore, the first and second finger as well as the fourth toe was not marked so as to avoid impacting their climbing abilities. Neither chorus tenure nor the rate of change in condition of males varied with the marking methods [46].

### Feeding experiments

During the main investigation in 2002, departing males, on their first night at the breeding aggregation, were provided with supplementary energy in the form of crickets. If a male had not left the breeding aggregation during the first night, it was fed on the second night. In 2003, the feeding experiments were carried out on only three consecutive days. Within seasons, males were not treated more than once.

For feeding experiments, males were put in small plastic boxes (10 × 10 cm) perforated for air supply and containing wet cellulose to prevent them from dehydration. The animals were divided into three groups: 1) Fed1 – males were offered one cricket, 2) Fed2 – males were supplied with 10 crickets, not all of which were eaten, 3) control males were not fed. Males were randomly assigned to one of the experimental groups. To increase sample size, animals that did not eat any of the offered crickets were later assigned to the control group (N = 6). All individuals were kept in the containers for 12 hours. Apart from the reassigned males, control males had no opportunity to feed while they were restrained.

For every fed male the amount and mass of the consumed crickets was determined. The average amount of energy contained in a gram cricket is 8033 J [25]. With an assimilation efficiency of 0.76 [25] a male tree frog acquires 5944 J/g digested cricket [47].

### Condition

If breeding behaviour is energetically costly then males should lose condition between their first and their last night in the chorus, unless they can compensate by increasing energy uptake. Furthermore, males with longer chorus tenure should have a higher initial body condition, a lower final condition or a lower rate of change in condition. To test these predictions, males were measured with a vernier calliper (measurement error: ± 0.1 mm) when they were first caught and body mass taken with a portable scale (Satorius Handy; measurement error: ± 0.01 g). Before weighing, males were put in a container with pond water to fully rehydrate them.

An Index of Condition (sensu [33,48]) was calculated for every night a male was weighed. A male's condition was determined by regression between the initial body mass of all males and their tibiafibula length. The initial condition of a male was defined as the deviation of its body mass from the predicted body mass by regression (residuals). The final condition was defined as the difference between the final body mass of a male on its last visit and the body mass calculated by regression (i.e. initial body mass to tibiafibula length). The rate of change in condition of a male was determined by the difference between the starting and the final condition divided by the number of nights between their first and last night in the breeding aggregation. All nights were counted, from the first to the final visit of a male in the chorus, irrespective of whether a male was present in the intervening period or not.

To determine if all males of each group had lost weight during their first and their final night at the breeding aggregation, only those animals were analysed that stayed at the breeding aggregation for more than one night after the feeding treatment. When measurement data of the final night were missing, the data of the last measurement was taken instead (N = 17).

### Chorus tenure

The chorus tenure of each male was recorded and compared between all groups. As the weather condition was different every night, this could have had an impact on the chorus tenure of the treated male. To avoid such seasonal effects, we paired Fed1, Fed2 and control males by night.

### Recording of calls

On several nights between 31st of May and 18th of June 2002 the rate of calling was recorded at the two study ponds. In addition, there were recordings at all four ponds between 24th of May and 14th of June 2003. Calls were recorded using several recorders (Sony Professional Walkman WM6DC and Marantz PMD 430) and microphones (Vivanco EM32) simultaneously. The recordings started after the breeding aggregation had settled and the calling sites/territories had been established. We moved slowly towards a group of calling males and recorded one or several cycles of calling. During a cycle, each male was recorded a minimum of 5 – 10 minutes. Males stopped calling only briefly when approached. Most recordings were made between 2300 and 0130. During recordings, the air and water temperature near calling males was measured by means of a digital thermometer.

The recordings were used to determine the calling rate of males. For statistical analysis of calling rate, the values of Fed1 and Fed2 males were pooled and compared to the control group using a paired design to control for variability in calling rate due to variation in chorus size (Meuche & Grafe unpublished, [49]) and temperature [49-51] factors known to strongly influence calling rate. To control the impact of these social and climatic factors, one control male and one fed male, which had been calling simultaneously, were paired. In addition, only those callers that were both calling in the same microhabitat (water or land) were compared. To avoid pseudoreplication, males were marked individually by toe-clipping [46].

Since the arrival of males was highly synchronized (Meuche & Grafe unpublished), it is unlikely that differences in calling rate between control and fed males are the result of different times of arrival. If not stated otherwise, means ± SD are given, and all tests are two-tailed.

## Authors' contributions

IM and TUG participated in design of the study and data collection. IM performed the statistical analysis and wrote the manuscript. TUG helped to draft the manuscript. All authors read and approved the final manuscript.

## Note

Table 1 – Changes in weight and condition

Medians of changes in weight and condition for Fed1, Fed2 and Control (K) males during the reproduction season in the year 2002.
